# Effect of Chelant-Based Soil Washing and Post-Treatment on Pb, Cd, and Zn Bioavailability and Plant Uptake

**DOI:** 10.1007/s11270-021-05356-0

**Published:** 2021-09-28

**Authors:** Christoph Noller, Wolfgang Friesl-Hanl, Rebecca Hood-Nowotny, Markus Puschenreiter, Andrea Watzinger

**Affiliations:** Department of Forest- and Soil Sciences, Institute of Soil Research, University of Natural Resources & Life Science (BOKU), Konrad-Lorenz Str. 24, 3430 Tulln, Austria

**Keywords:** Toxic metals, Remediation, Soil pollution, Zero-valent iron, Vermicompost, Biochar

## Abstract

The remediation of Pb, Cd, and Zn contaminated soil by ex situ EDTA washing was investigated in two pot experiments. We tested the influence of (i) 0, 0.5, 1.0, and 1.5%wt zero-valent iron (ZVI) and (ii) a combination of 5%wt vermicompost, 2%wt biochar, and 1%wt ZVI on the metal availability in EDTA-washed soil using different soil extracts (Aqua regia, NH_4_NO_3_) and plant concentrations. We found that EDTA soil washing significantly reduced the total concentration of Pb, Cd, and Zn and significantly reduced the Cd and Zn plant uptake. Residual EDTA was detected in water extracts causing the formation of highly available Pb-EDTA complexes. While organic amendments had no significant effect on Pb behavior in washed soils, an amendment of ≥ 1%wt ZVI successfully reduced EDTA concentrations, Pb bioavailability, and plant uptake. Our results suggest that Pb-EDTA complexes adsorb to a Fe oxyhydroxide layer, quickly developing on the ZVI surface. The increase in ZVI application strongly decreases Zn concentrations in plant tissue, whereas the uptake of Cd was not reduced, but even slightly increased. Soil washing did not affect plant productivity and organic amendments improved biomass production.

**Supplementary Information:**

The online version contains supplementary material available at 10.1007/s11270-021-05356-0.

## Introduction

Soils exposed to potentially toxic metal (PTM) immissions present a direct sink for those pollutants, since metals do not decompose and accumulate via atmospheric deposition (Adriano et al., 2004). Some PTM fractions can be transported over great distances and if mobilized cause biotoxicity (Hou et al., 2006). Soil contamination can be found all over the world, mostly stemming from mining activities. To remediate such contaminated sites, a large variety of technologies has been developed, among them soil excavation, washing, phytoremediation, or metal stabilization. While sustainable technologies have gained some support, their implementation is rare. Phytoremediation is still limited to areas where decontamination is not urgent (Suman et al., 2018). Immobilization approaches are generally effective and incur low costs, but the long-term stability of a system has to be monitored constantly to prevent future environmental risks (Lwin et al., 2018). Traditional dig and dump approaches, as well as physical separation, are therefore still widely used.

A promising sustainable treatment could be provided by a soil washing developed by Lestan (2017) known as ReSoil®. It uses (sodium-) ethylenediamine-tetraacetate (EDTA), known for its strong complexing properties to remove Cd, Pb, and other PTMs from multi-contaminated soils. Chelating agent-based soil washing shows the highest removal efficiency among remediation technologies but is also rather expensive due to the machinery and chemical costs (Dermont et al., 2008). Therefore, the washing solution is recycled in two steps. After separating the washing solution from the solid phase, EDTA-complexed PTMs are substituted using Ca(OH)_2_ and precipitated onto polysaccharides. EDTA is subsequently recovered using acid precipitation of Ca using H_2_SO_4_. Excess reagents are being removed as CaSO_4_. The resulting metal waste is marginal and the recirculation of the washing solution eliminates the need for expensive wastewater treatment. It has been demonstrated that the washing treatment significantly lowered total and plant-available metal fraction in alkaline and acidic soils (Lestan, 2017). On industrially contaminated soils, the procedure achieved extraction rates of 79, 70, and 38% for Pb, Cd, and Zn respectively (Voglar & Lestan, 2012), while plant growth was improved after the washing treatment (Jelusic & Lestan, 2015; Jelusic et al., 2014a, 2014b). The greatest obstacle so far has been the complete removal of EDTA from soil after PTM extraction, in order to prevent highly mobile metal-EDTA complexes as an environmental hazard. Gluhar et al. (2019) reported that even traces of EDTA after soil washing resulted in an increase in bioavailable PTMs. An amendment with zero-valent iron (ZVI) successfully reduced the PTM availability in acid and alkaline soils by adsorbing the PTM-EDTA complexes or precipitating them into an insoluble compound (Gluhar et al., 2019; Gylienė et al., 2008; Nowack, 2002). However, no plant trials have been performed to investigate the effects of increasing ZVI amendments on PTM uptake and plant growth.

In addition to the high extraction efficiency of EDTA washing, ex situ soil treatments technologies are the most invasive soil treatments (Koptsik, 2014). The additional chemical treatment further contributes to fundamental alteration of the soil physical, chemical, and biological properties (Jelusic et al., 2014a, 2014b; Kaurin et al., 2018; Maček et al., 2016). The complexation of metal cations by EDTA is non-selective and leads not only to the mobilization and subsequent extraction of PTMs but will also remove macro and micro nutrients (Dermont et al., 2008; Jelusic, et al., 2014a, 2014b). In the process, the detachment of metal ions from the soil matrix destabilizes soil minerals, oxyhydroxides, and the soil organic matter (SOM) (Ferraro et al., 2016; Tsang et al., 2007). This soil dissolution was observed by Lei et al. (2008) and Barona et al. (2001) and weakens the association of PTM with those soil components. It was suggested this could result in an even higher risk of plant uptake and ground water penetration compared to the untreated soil despite its higher total PTM content. Organic amendments like compost can be applied to ameliorate physical, chemical, and microbiological properties of such degraded soils. Especially vermicompost could serve as a bio-inoculum to restore microbiome of the soil after the invasive washing treatment (Lim et al., 2015). Biochar also has proven effective in ameliorating soil physical properties, improving the soil structure and water holding capacity of marginal soils. The synergetic effects of the two amendments have been studied broadly (D. Fischer & Glaser, 2012).

The present study describes the joint effects of EDTA washing and organic post-treatments on a soil contaminated by historic mining activities. It investigates the metal bioavailability and uptake of PTMs by radish plants (*Raphanus sativus L.* cultivar) in two pot experiments. The study addresses the negative impact on plant productivity after the remediation and the ameliorating effect of organic amendments. To assess the problem of mobile metal-EDTA complexes in the remediated soil, attendant potential health and environmental hazards, ZVI amendment was investigated as a possible sorbent for mobile complexes; the underlying mechanics of which will be discussed. Studying the effect of increasing ZVI amendments on plant growth and metal uptake will be a novelty for the Resoil® technology. The main hypotheses are that the washing treatment would lower the total PTM concentration below nationally recommended thresholds and ZVI will stabilize residual EDTA and thereby lower bioavailable PTM concentrations below the thresholds. Lastly, it was hypothesized that organic amendments would restore soil productivity and thus the biomass production on the washed soil.

## Material and Methods

### Soil

A heavily contaminated soil from Arnoldstein, Austria was used to investigate the effect of the EDTA soil washing. Therefore, Cambisol with loam texture (5–30 cm) from a pastureland (former arable land), close to the historic smelting complex, was excavated, after removing the grass layer. The soil washing and ZVI amendment were conducted by the group of Domen Lestan at the Biotechnical Faculty, University of Ljubljana, Slovenia. As described in detail by Gluhar et al., (2019), the contaminated soils were washed in batches of 50 kg using 60 mmol Na_2_-EDTA kg^−1^ solution (ratio 1:1 w/v) to extract PTMs. The washed soil used in both experiments aged for at least 2 months after the treatment. Soil washing changed the structure of the soil and the washed soil used in the pot experiments had an artificial macro structure developed during the washing process after passing the soil through a 5 mm sieve (Zupanc et al., 2014).

### EDTA Stabilization Using Zero-Valent Iron—1^st^ Experiment

ZVI was obtained from a local workshop close to the washing facility. It was added to the soil slurry shortly before the end of the washing procedure, aiming to immobilize the remaining EDTA while simultaneously retaining the high PTM extraction efficiency. ZVI was applied in treatments at increasing concentrations of 0, 0.5, 1.0, and 1.5%wt. Altogether, five treatments were investigated: the contaminated soil (C) and the washed treatments with increasing ZVI concentrations (ZVI0, ZVI0.5, ZVI1.0, ZVI1.5). The effect of ZVI was tested in a plant experiment in 1000 ml pots. Three replicates were set up in randomized blocks. After the pots were filled with air-dried soil, radish (*Raphanus sativus L.* cultivar French Breakfast) was sown into the pots and drip irrigated daily to maintain field capacity. Plants were grown under controlled greenhouse conditions (Online Resource Figure S3) with a 14 h light period and approximate temperatures of 25 °C for daytime and 20 °C for nighttime. Seedlings were thinned to three plants after germination and grown for 5 weeks.

### Soil Revitalization Using Organic Amendments—2^nd^ Experiment

In a second experiment, soils were amended with vermicompost and biochar to investigate their effects on the soil fertility and metal retention (Paz-Ferreiro et al., 2014). Three different soils were investigated: The contaminated soil (C) and two washed soils (W, WZ), the second one amended with 1%wt ZVI. The ZVI concentration was chosen after evaluating the results of the first experiment. All soils were amended with 5%wt vermicompost (Vermigrand Naturprodukte GmbH) and 2%wt biochar pyrolyzed at 600 °C (Sonnenerde GmbH), based on dry weight (CA, WA, WZA). Since washed soils are known for low water holding capacities (Zupanc et al., 2014), the biochar amendment was aiming to improve the physical soil properties due to the high specific surface (Online Resource Table S2) and test its effect on plant growth before its field application. Compost was applied as a further organic fertilizer to create a soil suitable for gardening purposes (Online Resource Table S1) and to compensate possible nutrient retention by the biochar. The experiment was conducted using four replicates in randomized blocks. Pots were filled with air-dried soil (550 ml) and radish (*Raphanus sativus L.* cultivar French Breakfast) was sown. Plants were grown under the same greenhouse conditions used in the first experiment. Again, seedlings were thinned to three plants after germination and grown for 5 weeks until radish bulbs were developed.

At harvest, roots and leaves were separated, washed, weighed, and dried at 60 °C until a constant weight was reached. The plant tissue was ground in a stainless-steel ball mill prior to acid digestion. Due to insufficient bulb material in the C treatments, the aerial tissue was used for evaluation PTM plant uptake. Soil samples were taken immediately after the harvest, air dried, sieved using a < 2 mm stainless-steel mesh, and stored in plastic bags until further analysis. For acid digestion, soil samples were finely ground in a stainless-steel ball mill.

### Soil and Plant Analysis

Gravimetric water content was determined using 5 g of soil dried at 105 °C and all the results were converted and reported on a dry weight basis. The soil pH was determined in deionized water and 0.01 M CaCl_2_ using a pH meter (w/v ratio 1:2.5) (ÖNORM L 1083:^2005^). The total carbon and nitrogen contents were determined using ground plant or soil samples by Dumas combustion (Dumas, 1826 as cited by Buckee, 1994) elemental analysis (Flash 2000, Thermo Scientific). Cation exchange capacity was determined in a 0.1 M BaCl_2_ extract (w/v ratio 1:20)(ÖNORM L 1086–1:^2014^) by ICP-OES (Optima 8300, Perkin Elmer). Potentially plant-available metals were measured following extraction with 1 M NH_4_NO_3_ (w/v ratio 1:2.5)(DIN 19,730:^1993^). The total metal concentration was determined after aqua regia digestion of 0.5 g of soil (1.5 ml HNO3 65%, 4.5 ml HCl 37%) in a heating block at 150 °C for 3 h (ÖNORM L 1085:^2013^). Plant metal content was measured after acid digesting of 0.2 g sample (5 ml HNO_3_ 65%, 1 ml H_2_O_2_ 30%) in a heating block at 155 °C for 2.5 h. Digestion vials were equipped with coolers.

Pb and Cd were measured by GF-AAS (HGA 900 coupled with AAnalyst 400, Perkin Elmer) and Zn by F-AAS (AAnalyst 400 Flame, Perkin Elmer), both with deuterium lamp background correction. Five µL ammonium hydrogen phosphate was used as a modifier in a 1:4 ratio to decrease matrix effects (Schlemmer, 2013; Viñas et al., 1997). Blank samples, quality control standards, certified reference material (Plants: INCT-OBTL-5, Soil: ISE 885) were included in each run. Recovery rates ranged between 88 and 110%. The limit of detection was 0.4 µg L^−1^, 0.05 µg L^−1^, and 2.2 mg L^−1^ for Pb, Cd, and Zn, respectively.

EDTA was measured in water extracts (1:2 w/v ratio, shaking time 2 h) following an adapted spectrometric method by Wang et al. (2013), an indirect measurement using ferric iron. The pH of 15 ml water extract was raised to a pH 10 using 1 M NaOH. At pH > 7, EDTA gets unstable and is expected to be present in its uncomplexed state. To oxidize interfering dissolved organic carbon (DOC), the method was adapted by the addition of 0.1 g MnO_2_ powder. After 10 min, the precipitated Fe was removed by filtration (Whatman, syringe filter, Cellulose Acetate, 0.45 µm). Eighty-six µL of sample solution was added to a 2 ml Eppendorf vial together with 10 µL of 4 mM ammonium ferric sulfate (AFS, adjusted to pH 1) and 5 µM of 2 M HCl (check if pH is ≤ 2) and incubated for 10 min at room temperature. During this time, Fe^3+^ forms complexes with EDTA. To reduce excess Fe^3+^ to Fe^2+^, 35 µL of 45 mM Na_2_SO_3_ was added and incubated 10 min at room temperature. Then, 20 µL of 1,10-phenanthroline monohydrate and 75 µL of 2 M NaAc buffer (pH 4.7) were added to the vials. The samples were briefly shaken by hand, pipetted into microtiter plates and measured at 510 nm after 20 min (Enspire 2300, Perkin Elmer). A standard sequence (5–50 µM) was prepared from 100 µM AFS stock solution and verified by a second calibration using single element ICP iron standard solution (1000 mg L^−1^, Certipur®, Merck). The recovery of AFS and EDTA ranged from 90–98%, tested in blanks and soil extracts.

### Statistical Analysis

The non-parametric Krushkal-Wallis test and Dunn’s multiple comparison post hoc test was used to determine differences between the PTM concentrations in soil extracts and plant material. Correlation analysis was performed using Spearman’s rank correlation. Significance was assessed at the 5% level throughout. The statistical analysis was carried out using R studio version 1.1.423 (Allaire, 2012; R. Core Team, 2013) and figures were plotted using the package ggplot2 (Wickham, 2011).

## Results and Discussion

Soil washing significantly increased soil pH (Tables 1 and 2). This can be attributed to the formation of CaSO_4_ in the washing process. The application of biochar and vermicompost further contributed to this effect. The increasing ZVI amendment (0.5%, 1.0%, 1.5%) had no significant effect on the soil pH.

**Table 1:** Soil chemical analysis of the soils used in the EDTA stabilization experiment. *C* contaminated soil, *ZVI0/0.5/1.0/1.5* washed soil amended with 0, 0.5, 1.0, 1.5%wt ZVI. Means ± standard deviation (*n* = 3). Lower case letters represent significant differences between homogeneous groups. Austrian thresholds in mg kg^−1^ for Aqua regia: Pb 100, Cd 0.5, Zn 300. NH_4_NO_3_: Pb 0.3, Cd 0.04, Zn 4.0

Property	C	ZVI0	ZVI0.5	ZVI1.0	ZVI1.5
pH	4.94 ± 0.06_a_	6.08 ± 0.05_b_	6.12 ± 0.05_b_	6.09 ± 0.04_b_	6.23 ± 0.04_b_
EDTA (µg kg^−1^)	**─**	912 ± 40_a_	151 ± 31_b_	131 ± 34_b_	89.2 ± 12.8_b_
Aqua regia (total)					
Pb (mg kg^−1^)	882 ± 33_a_	208 ± 11_b_	205 ± 4_b_	194 ± 8_b_	199 ± 11_b_
Cd (mg kg^−1^)	5.12 ± 0.05_a_	1.84 ± 0.17_b_	1.81 ± 0.07_b_	1.74 ± 0.05_b_	1.64 ± 0.10_b_
Zn (mg kg^−1^)	474 ± 13_a_	342 ± 9_b_	334 ± 6_b_	326 ± 7_b_	341 ± 7_b_
Ammonium nitrate (bioavailable)					
Pb (mg kg^−1^)	8.29 ± 0.70_b_	12.3 ± 2.70_a_	0.35 ± 0.11_c_	0.15 ± 0.03_d_	0.07 ± 0.02_e_
Cd (mg kg^−1^)	0.85 ± 0.085_a_	0.343 ± 0.083_b_	0.049 ± 0.01_c_	0.033 ± 0.003_d_	0.024 ± 0.003_e_
Zn (mg kg^−1^)	34.2 ± 5.3_a_	12.7 ± 2.1_b_	1.42 ± 0.37_c_	0.89 ± 0.08_d_	0.52 ± 0.06_e_

**Table 2:** Soil chemical analysis of the soils used in the revitalization experiment. C contaminated soil, *CA* contaminated amended with 5%wt vermicompost and 2%wt biochar. *W* washed soil, *WZ* washed soil amended with 1%wt ZVI, *WA* washed soil amended with 5%wt vermicompost and 2%wt biochar. *WZA* washed soil amended with 1%wt ZVI, 5%wt vermicompost and 2%wt biochar. Means ± standard deviation (*n* = 4). Lower case letters represent significant differences between homogeneous groups. Austrian thresholds in mg kg^−1^ for Aqua Regia: Pb 100, Cd 0.5, Zn 300. NH_4_NO_3_: Pb 0.3, Cd 0.04, Zn 4.0

Analysis	C	CA	W	WA	WZ	WZA
pH	5.14 ± 0.21_a_	6.67 ± 0.12_c_	6.15 ± 0.12_b_	6.86 ± 0.03_c_	6.84 ± 0.07_c_	7.17 ± 0.07_d_
EDTA (µM kg^−1^)	**─**	**─**	275 ± 21_a_	118 ± 11_b_	23.5 ± 0.9_c_	25.6 ± 5.3_c_
Aqua regia (total)						
Pb (mg kg^−1^)	885 ± 27_a_	882 ± 37_a_	238 ± 8_b_	220 ± 12_b_	149 ± 19_c_	152 ± 7_c_
Cd (mg kg^−1^)	4.87 ± 0.14_a_	4.96 ± 0.36_a_	1.44 ± 0.01_b_	0.99 ± 0.04_b_	1.07 ± 0.04_b_	1.32 ± 0.07_b_
Zn (mg kg^−1^)	449 ± 9_a_	425 ± 36_a_	282 ± 7_b_	299 ± 9_b_	286 ± 17_b_	347 ± 11_b_
Ammonium nitrate (bioavailable)						
Pb (mg kg^−1^)	13.1 ± 2.3_a_	0.19 ± 0.03_c_	5.16 ± 1.32_b_	7.24 ± 1.62_b_	0.05 ± 0.01_d_	0.02 ± < 0.01_e_
Cd (µg kg^−1^)	850 ± 120_a_	34.6 ± 4.9_d_	110 ± 20_c_	210 ± 40_b_	9.54 ± 0.69_e_	4.72 ± 0.12_f_
Zn (mg kg^−1^)	29.5 ± 2.8_a_	0.33 ± 0.07_c_	1.80 ± 0.24_b_	2.22 ± 0.54_b_	< 0.05_d_	< 0.05_d_

### Trace Metal Extraction by Soil Washing

Prior to remediation, the soil was heavily contaminated with PTM, with total values of 885 mg kg^−1^ Pb, 4.87 mg kg^−1^ Cd, and 449 mg kg^−1^ Zn. Washing with EDTA removed a significant portion of the PTMs, reducing the total concentrations in WZ of Pb, Cd, and Zn to 149, 1.07, and 286 mg kg^−1^ respectively (Table 2). Most of the remaining metals persist in forms inaccessible to plants but it is possible that residual available metal fractions were present in the remediated soil (Jelusic & Lestan, 2014). The relatively low reduction of total Zn concentration is related to its association with Fe-/Al-oxides and the residual fraction, inaccessible to EDTA (Adriano, 2001; Jelusic et al., 2014a, 2014b).

The efficiency of EDTA soil washing was initially assessed by comparing the total concentrations of the relevant PTM (Pb, Cd, Zn), providing an initial estimate of soil contamination (Table 1 and 2). Except for Zn the unamended remediated soils (ZVI0, W, WZ) fell within general guideline values of Germany and Finland (200, 10, 250 mg kg^−1^ for Pb, Cd, Zn), common in the European Union (BMJV 1999; Ministry of the Environment, 2007), but did not match the standards of the Austrian Government, specifically those for soils used for vegetable production: 100, 0.5, 300 mg kg^−1^ for Pb, Cd, and Zn (ÖNORM S 2088–2:^2014^). Furthermore, it has been suggested that total soil concentrations are not an appropriate measure for a comprehensive assessment of ecological risks or risks to human health (Gupta et al., 1996). Even if the complete removal of the phytoavailable fraction can be expected, a resupply of previously stable metal ions into more labile fractions, due to a change in the equilibrium of the solid–liquid phase concentration is possible (Udovic & Lestan, 2010). The labile fraction, as well as actual plant uptake, should also be taken into account. NH_4_NO_3_ extractable metals are commonly used as an approximation of the bioavailable fraction. The extract contains the mobile and potentially plant-available metals and encapsulates the influence of important soil properties like contents of clay, SOM, oxyhydroxides, and the soil pH (Gupta et al., 1996) and was therefore adopted to this experiment as a proxy of bioavailable fraction. The concentration of Cd and Zn in the bioavailable fraction was significantly decreased in all EDTA-washed treatments, while Pb only was decreased in treatments spiked with ZVI. The PTM concentration fell below Austrian thresholds (0.3, 0.04, 4 mg kg^−1^ for Pb, Cd, Zn), specifying extractable metal concentrations, but only in treatments with ZVI ≥ 1%wt (ZVI1.0, ZVI1.5, WZ, WZA) (ÖNORM S 2088:^2014^).

### EDTA Stabilization by ZVI: 1st Experiment

**Fig. 1:**
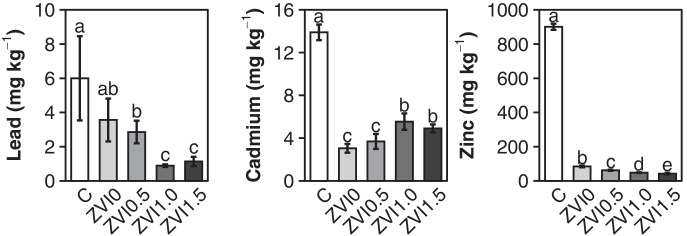
Metal concentrations per dry weight in the leaf tissues of radish plants. C contaminated soil, ZVI0/0.5/1.0/1.5 washed soil amended with 0, 0.5, 1.0, 1.5%wt ZVI. Lower case letters represent statistically homogenous groups

In plants grown on the contaminated soil, tissue concentrations of 6.0 mg kg^−1^ Pb, 13.4 mg kg^−1^ Cd, and 900 mg kg^−1^ Zn were measured. Soil washing had a significant effect on the PTM bioavailability and plant uptake (Table 1, Fig. 1) and reduced the plant uptake to 0.89 mg kg^−1^ Pb, 5.5 mg kg^−1^ Cd, and 48 mg kg^−1^ Zn for the 1% ZVI-amended treatment. The bioavailable fraction of Cd and Zn in soil and their uptake by the plants decreased in all treatments, although Pb only decreased if ZVI was added. High concentrations of mobile Pb present after in the washed soils are attributed to residual EDTA (Table 1) and the formation of mobile Pb-EDTA complexes (Gluhar et al., 2019). The formation constant of metal-EDTA complexes indicates that Pb (log *K* = 18.0, measured at 25 °C and 0.1 mg ml^−1^ ionic strength) is generally favored over Zn (log *K* = 16.5) and Cd (log *K* = 16.5) in a range of pH 2–9 (Begum et al., 2012; Martell & Smith, 1974), explaining the discrepancy between the high plant uptake of Pb compared to Cd and Zn in the presented experiments. While it is reasonable to believe that EDTA complexes do not adsorb to mineral surfaces, considering they are used to keep metals dissolved, this is only true for chelating agents used extensively for technical applications (Nowack, 2002). If present in low concentration, EDTA complexes can even increase metal sorption significantly (Davis & Leckie, 1978). Using FTIR data from a sorption experiment, Tsang and Hartley (2014) concluded that certain chelating agents are highly localized on Fe oxides. Considering further fundamental publications on EDTA(-complex) behavior in solution (Bowers & Huang, 1986; Nowack, 2002; Zachara et al., 1995), it can be assumed that Pb-EDTA adsorption is limited to protonated surface hydroxyl groups found on Fe oxyhydroxides (Cornell & Schwertmann, 2003). Under neutral to acidic pH and oxic soil conditions, it can be expected that the ZVI added to the soil undergoes a rapid oxidization to Fe^2+^ and Fe^3+^ creating a shell around the zero-valent core (Dorjee et al., 2014). The oxidized shell is further transformed by hydrolyzation to weakly crystallized ferric hydroxides Fe(OH)_3_ and oxyhydroxides FeOOH (Cornell & Schwertmann, 2003). Most Fe oxyhydroxides share a high point of zero charge, owing a net positive charge from acidic to slight alkaline pH (Blume et al., 2016). They are therefore ideal sorbents of negatively charged metal-EDTA complexes (Davis & Leckie, 1978). The character of the complex adsorption is assumed to be either electrostatic or occurring as a type B ternary surface complex with a ligand-like adsorption behavior (Bourg & Schindler, 1978; Bowers & Huang, 1986). In the latter case, EDTA forms a bridge between the metal and the Fe oxide surface (Nowack, 2002). We suspect this interaction between ZVI and metal-EDTA complexes was the driving factor behind the decrease in water-soluble EDTA in all ZVI-amended soils (Table 1) and the reduction of Pb uptake into plants grown on ZVI0 and ZVI1.0 from 3.8 to 0.89 mg kg^−1^.

**Fig. 2:**
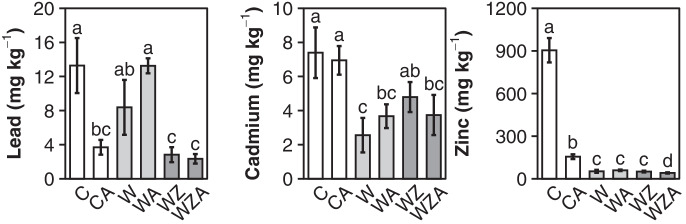
Metal concentrations per dry weight in the leaf tissues of radish plants. C contaminated soil, CA contaminated amended with 5%wt vermicompost and 2%wt biochar. W washed soil, WA washed soil amended with 5%wt vermicompost and 2%wt biochar. WZ washed soil amended with 1%wt ZVI, WZA washed soil amended with 1%wt ZVI, amended with 5%wt vermicompost and 2%wt biochar. Lower case letters represent statistically homogenous groups

**Fig. 3:**
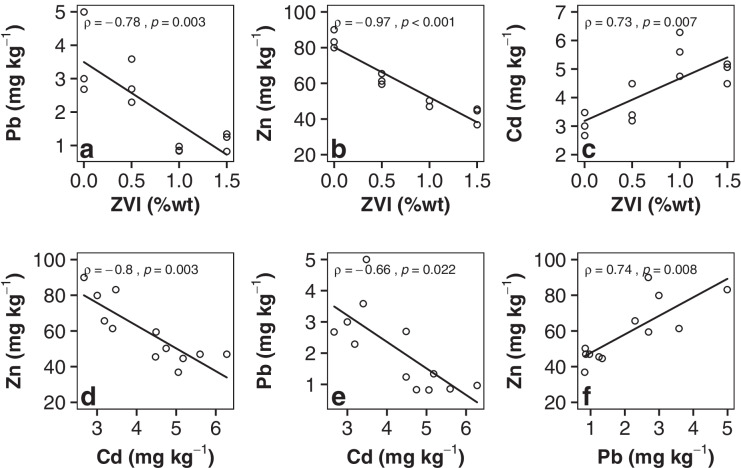
The figures show data from the 1^st^ experiment.** a–c** Show the linear dependence of PTM concentrations in radish leave tissue and ZVI soil amendment. **d**–**f** Show the linear relation between the plant uptake of single PTMs into radish leave tissue. The Spearman correlation coefficient (*ρ*) and the statistical significance (*p*) are indicated

The amount of ZVI applied during the washing procedure was essential in lowering the metal phytoavailability. Each increment in ZVI application significantly lowered the fraction of bioavailable Pb, Cd, and Zn (Table 1). The Zn plant uptake strongly decreases after soil washing without further amendment, possibly due to a high extraction efficiency of plant-available fractions and a significant pH raise. Each further ZVI increment translated into even lower plant uptake (Figs. 1 and 2) because its sorption to Fe oxides is especially crucial at slightly acidic pH (Moreno-Lora & Delgado, 2020). A significant reduction in plant Pb was only reached after substantial ZVI amendment (treatments ZVI1.0 and ZVI1.5) and explained by the sorption of EDTA-Pb complexes. Cd behaved in the opposite manner. ZVI application and Cd plant uptake showed a positive correlation in the presented data (Fig. 3c) contrasting the decreasing bioavailable concentrations (Table 1). Until now, the only study investigating the effect increasing ZVI concentrations on PTM in soil treated by EDTA washing only used an equilibrium-based soil extraction to assess its availability and did not find a positive correlation with Cd plant uptake (Gluhar et al., 2019). The lowest Cd concentration in plant tissue is found in the ZVI-free treatment and significantly increased in the ZVI1.0 and ZVI1.5 treatments (Fig. 1). In a previous plant experiment, the same EDTA-washed soil was investigated by Kaurin et al. (2020) and NH_4_NO_3_ extractable PTMs were measured by Gluhar et al. (2020) using a fixed ZVI level of 1% while no ZVI-free treatment was included. The results showed significantly lower Cd uptake by buckwheat (F. esculentum, 1.5 mg kg^−1^) and Chinese cabbage (B. rapa, 0.6 mg kg^−1^) compared to concentrations in radish plants (*Raphanus sativus L*) presented here (WZ: 4.8 mg kg^−1^, ZVI1.0: 5.5 mg kg^−1^). Anaerobic conditions during the experiment were considered to explain this difference, since they are common in pot experiments and could hinder Cd sorption to ZVI due to competition with increasing levels of Fe in the soil solution, but since bioavailable PTMs match closely and Fe levels are decreased after ZVI amendment (Online Resource Table S3), the discrepancy in Cd uptake could merely be plant-specific. Other studies on ZVI soil amendments reported a decrease in Cd bioavailability as well as plant uptake (Jiang et al., 2018). However, an increasing Cd uptake after ZVI amendment is not a unique observation. Agreeing with our findings, Gong et al., (2017) demonstrated that (modified) ZVI can increase Cd plant uptake. This inefficiency of ZVI to stabilize PTMs under acidic soil conditions is still being investigated (Vítková et al., 2017) but could attribute to the pH dependent sorption behavior of Cd (Serrano et al., 2009). Gil-Díaz et al. (2017) and Serrano et al. (2005) showed that under acidic conditions Cd does not compete effectively for variable charged surfaces in the presence of other metals due to its lower tendency to form hydrolysis products (L. Fischer et al., 2007; Srivastava et al., 2005) and a slightly lower standard potential E^0^ compared to oxidized ZVI (Li & Zhang, 2007). This means that Cd is more dependent on electrostatic interactions with exchange sites, while Pb prefers covalent bounds with mineral complexes. Loganathan et al. (2012) summarize that in multi-contaminated soils, other PTMs like Zn and Pb can significantly reduce the sorption of Cd to oxides, SOM, and clay minerals. Additionally, considering the negative correlation between Pb/Zn and Cd in plant tissue (Fig. 3d–e), the increasing Cd uptake could stem from the antagonistic relationship between Cd to other cations like Pb, Zn, Ca, or Na, introduced during the washing and shown in the supplementary data (Online Resource Table S3). In a study comparing the dose–response of single and binary metal mixtures, Sharma et al. (1999) found that Zn antagonized the Cd plant uptake at concentrations similarly to this study. Under the present conditions, ZVI is not effective and even counterproductive in lowering Cd plant uptake.

### Revitalization of Washed Soil: 2nd Experiment

The concentrations of PTMs in plants grown on the contaminated soil were 13.2 mg kg^−1^ Pb, 7.39 mg kg^−1^ Cd, and 905 mg kg^−1^ Zn and were reduced to 2.83 mg kg^−1^ Pb, 4.79 mg kg^−1^ Cd, and 50 mg kg^−1^ Zn by soil washing with ZVI application (WZ). Except for Cd, the organic amendment further reduced bioavailability and plant uptake significantly (WZA) (Table 2, Fig. 2). Bioavailable metals and concentrations in plant tissues did not correlate well with the measured soil pH (Online Resource Table S4, S5). In the washed treatment (W), bioavailable Pb was significantly reduced (Table 2) but still at a high level compared to the ZVI-amended treatments (WZ, WZA) and had no significant effect on Pb plant uptake (Fig. 2).

The organic amendment significantly lowered Pb bioavailability and plant uptake in the contaminated treatment (CA) (Table 2, Fig. 2). Vermicompost and biochar had no significant effect on Pb behavior in the washed soil (WA). This was found to be due to the presence of highly mobile Pb-EDTA complexes mentioned earlier, which cannot be adsorbed by the SOM. Like in the CA treatment, vermicompost and biochar have successfully been used for PTM stabilization in the past (Paz-Ferreiro et al., 2014; Zhou et al., 2017). This is attributed to carboxylic and phenolic functional groups (highly affine to Pb^2+^) which possess a negative charge under acidic soil conditions due to rapid deprotonation (Blume et al., 2016). At slightly acidic soil pH (Table 2), EDTA exists as a divalent negative charged complex Pb-EDTA^2−^ (Harris, 2015; Krishnan et al., 2003) and is generally repelled by SOM since positively charged functional groups like amines contribute much less to the total charge (Strawn et al., 2020). The surface properties of biochars are very heterogeneous and mainly determined by feedstock, production parameters, and pos-treatment (Uchimiya et al., 2011). Freshly produced biochars are characterized by a net positive surface charge (Cheng et al., 2008), especially for chars produced at higher temperatures (> 500 °C) (Banik et al., 2018). Nevertheless, in this experiment, their addition had no effect on the Pb-EDTA bioavailability since the total amount of functional groups was very low compared to the SOM added (Lehmann, 2007). The fact that Pb uptake was only decreased in the washed treatments amended with ZVI illustrated again that metal-EDTA complexes mostly absorbed to Fe-oxyhydroxides agreeing with the findings from the EDTA stabilization experiment.

The bioavailable fractions of Pb, Cd, and Zn in the contaminated soil (CA) were significantly reduced by the vermicompost and biochar amendment also translating to lower plant uptake except for Cd (Fig. 2). In both experiments, Cd was a special case. Without the ZVI addition, soil washing significantly decreased Cd bioavailability and plant uptake in W and WA. Despite a much lower bioavailable fraction (Table 2) in the ZVI-amended soil (WZ), plant Cd increased significantly compared to W (Fig. 2). The organic amendment (WZA) partially reversed this increase. These observations are similar to findings from the EDTA stabilization experiment, were increasing ZVI loads reduced Pb and Zn plant uptake but were positively correlation with the Cd plant tissue concentration (Fig. 3c), although bioavailable Cd continuously decreased (Table 1). NH_4_NO_3_ extractable Cd shows no significant correlation with the plant uptake (Online Resource Figure S1), something that has been observed before in neutral soils (Hall et al., 1998; Karer et al., 2015). Especially the discrepancy between the bioavailable Cd in the CA treatment and Cd plant uptake stood out (Table 2, Fig. 2). It can be assumed that the vermicompost amendment leads to an increase in dissolved organic carbon raising the solubility and plant uptake of Cd (Egene et al., 2018) which is not always represented by the NH_4_NO_3_ extractable fraction (Shan et al., 2010). The high bioavailability may, however, also be explained by fast desorption rates of Cd: in a liming experiment, Smolders et al. (2020) found that liming hardly reduced plant Cd uptake, although a clear decrease in a soil extract was found. This shows that equilibrium-based extraction approaches may suffer from limited predictability of plant metal uptake.

The strong decrease in Zn bioavailability and plant uptake in all treatments was due to Zn’s sensitivity to increase in pH (Fig. 2, Table 2) (Marschner, 1993) as discussed for the first experiment. It can be explained as an artefact of both the washing procedure and the organic amendments. In contrast to Cd, desorption rates for Zn are much lower (Smolders et al., 2020), which also likely contributed to the lower uptake of Zn by plants, when compared to Cd.

### Plant Growth

The artificial macro aggregates generated by the washing procedures appeared to allow for better soil aeration and development of the radish bulb. Plants germinated faster in most of the washed treatments (from observation). The contaminated soil had a higher bulk density in prior experiments (Zupanc et al., 2014), possibly to disturbance in the course of the pot experiment preparation, resulting in problems for the seedlings to penetrate the surface.

**Fig. 4:**
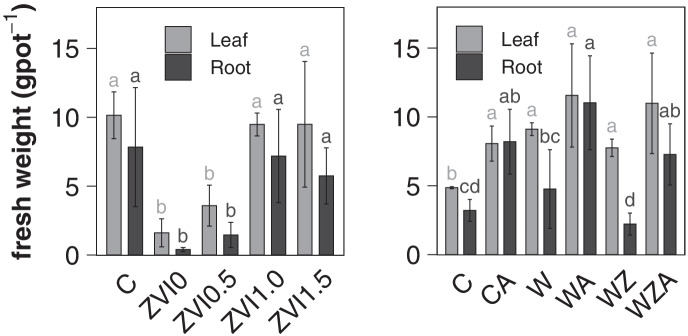
Biomass data from the EDTA stabilization experiment (left, *n* = 3) and the revitalization experiment (right, *n* = 4). The error bars represent the mean ± standard deviation. C contaminated soil, ZVI.0/0.5/1.0/1.5 washed soil amended with 0, 0.5, 1.0, 1.5%wt ZVI. C contaminated soil, W washed soil, WZ washed soil amended with 1%wt ZVI, A amended with 5%wt vermicompost and 2%wt biochar. Lower case letters represent statistically homogenous groups, separately tested for leaf and root data

The biomass of the radish plants showed high replicate variation as well individual variation within single pots. Soil washing did not have a clear positive effect on plant growth (Fig. 4). In the EDTA stabilization experiment, the soil washing leads to a significant decrease in leaf and bulb biomass for the treatments ZVI0 and ZVI0.5 when compared to the contaminated soil-C (Fig. 4). With increasing ZVI addition, the biomass increased back to values found in the contaminated C soil. Therefore, 1%wt ZVI was used in the rehabilitation experiment. While the high biomass production on the contaminated soil seems to be counterintuitive, it showed that the limiting factor for plant growth was not necessarily the high PTM concentration. Especially in the ZVI0 and ZVI0.5, the additive effect of high PTM mobility and biotoxicity of EDTA lead to growth depression (Grčman et al., 2001). Radish plants exposed to Pb toxicity are known to exhibit growth depression (Khan & Frankland, 1983), and in this study, Pb concentrations in plants grown on the washed treatments (excluding C) showed a strong negative correlation with biomass production (Online Resource Figure S2).

In the rehabilitation experiment (2^nd^ Experiment), soil washing did not result in a decrease in bulb biomass; instead, it increased the leaf biomass in all treatments significantly (Fig. 4). The remediation did change not only the PTM availability but also the soil structure. Radish bulb biomass production was lower in the non-fertilized treatments, clearly due to lower nutrient availability. The organic amendments significantly increased bulb biomass in the contaminated and washed treatments CA, WA, WZA.

## Conclusion

The efficient removal of PTM by EDTA soil washing has been proven before. However, the sustainability of the procedure depends on the complete removal or stabilization of residual EDTA in the remediated soil and the rehabilitation of soil functions for plant production. EDTA soil washing significantly reduced total Pb, Cd, and Zn soil concentrations. The results of two pot experiments also showed that traces of EDTA will form complexes with Pb due to their favorable formation constant leading to high plant uptake even at low total soil concentration. The application of ≥ 1%wt of ZVI was effective in stabilizing residual EDTA complexes and successfully reduced PTM plant concentrations to substantially lower levels compared to the contaminated soil but failed to match national regulation thresholds. Despite substantially reduced plant concentrations of Cd, increasing ZVI application led to a significant increase in Cd plant uptake compared to the EDTA-washed soil without the amendment which was in strong contrast to the bioavailable fraction determined by NH_4_NO_3_ extraction. NH_4_NO_3_  was an unreliable method to predict bioavailable Cd, possibly due to its higher kinetic lability and resupply from the solid phase compared to Zn and Pb. Organic amendments lowered the Cd plant uptake in EDTA-washed soils. Soil washing did not result in general loss in soil fertility and organic amendments contributed to an increased in plant yield. In the next stage, the washed soil will be tested in a field trial to investigate soil physical parameters, the midterm stability of the PTM-EDTA complexes, and PTM plant uptake under more realistic conditions.

## Supplementary Information

Below is the link to the electronic supplementary material.

Supplementary file1 (DOCX 384 KB)

## Data Availability

The datasets generated during and/or analyzed during the current study are available from the corresponding author on reasonable request.
