# Supplementary material for: Effect of Chelant-Based Soil Washing and Post-Treatment on Pb, Cd, and Zn Bioavailability and Plant Uptake
Source: Water Air Soil Pollut. 2021 Sep 28;232(10):405. doi: 10.1007/s11270-021-05356-0 (PMC8550514; doi:10.1007/s11270-021-05356-0)
Supplement: Supplementary file 1 — Supplementary file1 (DOCX 384 KB) [file 11270_2021_5356_MOESM1_ESM.docx]

**SUPPLEMENTARY MATERIAL for**

**Effect of chelant based soil washing and post treatment on Pb, Cd and Zn bioavailability and plant uptake**

Christoph Noller*_a_, Wolfgang Friesl-Hanl_a_, Rebecca Hood-Nowotny_a_, Markus Puschenreiter_a_, Andrea Watzinger_a_

_a_University of Natural Resources & Life Science (BOKU), Department of Forest- and Soil Sciences, Institute of Soil Research, Konrad-Lorenz Str. 24, 3430 Tulln, Austria

***Corresponding author**
christoph.noller@boku.ac.at

| **Legend** | | **Page** |
| --- | --- | --- |
| Figure S1 | Correlation between bioavailable Cd and plant uptake | 1 |
| Figure S2 | Correlation between biomass and Pb uptake | 1 |
| Figure S3 | Experimental set up of the 2^nd^ Experiment | 2 |
| Table S1 | Vermicompost description | 2 |
| Table S2 | Biochar description | 2 |
| Table S3 | BaCl_2_ extract | 3 |
| Table S4 | Correlation between Pb, Cd, Zn and H^+^ in experiment 1 | 3 |
| Table S5 | Correlation between Pb, Cd, Zn and H^+^ in experiment 2 | 3 |


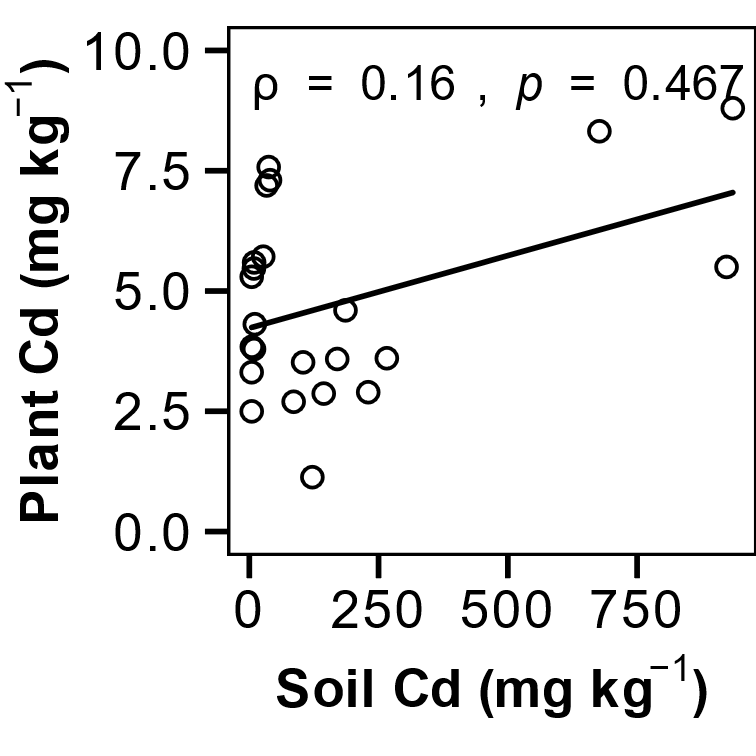


**Fig S1** Dependence between Cd leaf concentration and NH_4_NO_3_ extractable Cd for the 2^nd^ experiment. The Spearman correlation coefficient (ρ) and the statistical significance (p) are indicated.


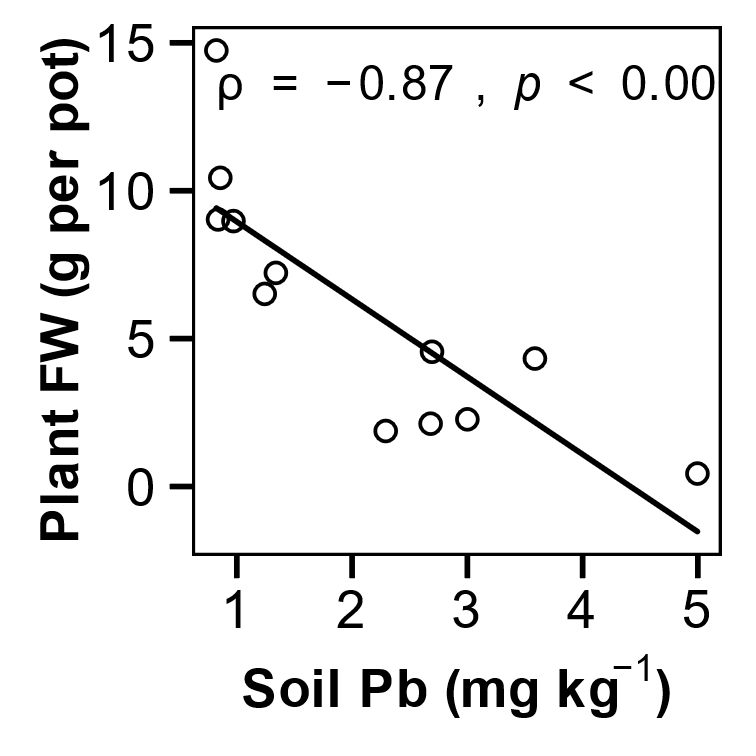


**Fig S2** Dependence between leaf fresh weight (g) and Pb plant uptake (mg kg^-1^) in the 1^st^ experiment. The Spearman correlation coefficient (ρ) and the statistical significance (p) are indicated.


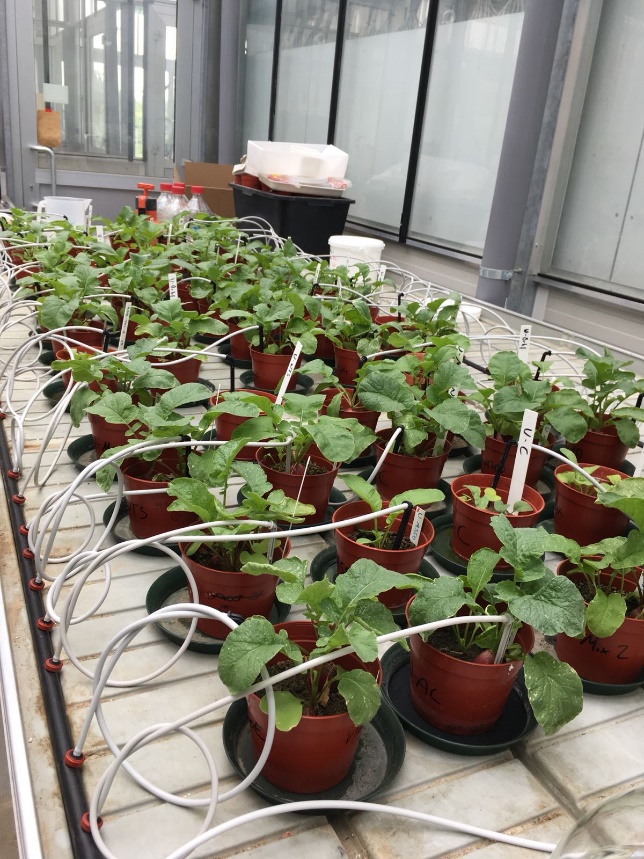


**Fig S3** Experimental set up of the 2^nd^ pot experiment.

**Table S1** Composition of the vermicompost amendment provided by the production company (Vermigrand Natruprodukte GmbH).

|  | Organic C | Total N | P_2_O_5_ | K_2_O | MgO | CaO |
| --- | --- | --- | --- | --- | --- | --- |
|  | g kg^-1^ | g kg^-1^ | g kg^-1^ | g kg^-1^ | g kg^-1^ | g kg^-1^ |
| Vermicompost | 310 | 14 | 5.0 | 14 | 20 | 10 |

**Table S2** Composition of the biochar amendment provided by the production company (Sonnenerde GmbH). Nutrients in biochar are mainly present in forms unavailable to plants.

|  | Organic C | Total N | H/C_org_ ratio | P_2_O5 | K_2_O | MgO | Surface area |
| --- | --- | --- | --- | --- | --- | --- | --- |
|  | g kg^-1^ | g kg^-1^ |  | g kg^-1^ | g kg^-1^ | g kg^-1^ | m^2^ g^-1^ |
| Biochar | 632 | 9.9 | 0.17 | 56 | 50 | 31 | 297 |

**Table S3** Data from a 0.1 M BaCl_2_ soil extraction (1:20 w/v ratio), analyzed with ICP-OES. Data represents mean ± standard deviation.

|  | Ca | Fe | K | Mg | Na | CEC |
| --- | --- | --- | --- | --- | --- | --- |
|  | mg kg^-1^ | | | | | cmol kg^-1^ |
| U | 1032 ± 24 | 0.83 ± 0.36 | 43.9 ± 6.9 | 216 ± 5 | 62.0 ± 8.3 | 7.3 ± 0.2 |
| W | 2021 ± 31 | 20.1 ± 1.68 | 102 ± 36 | 146 ± 4 | 179 ± 17 | 12.4 ± 0.3 |
| WZ | 2304 ± 77 | 2.54 ± 1.08 | 65.2 ± 11.8 | 162 ± 7 | 372 ± 32 | 14.6 ± 0.5 |
| WZA | 3050 ± 85 | 3.06 ± 1.97 | 595 ± 26 | 259 ± 12 | 300 ± 28 | 20.2 ± 0.4 |

**Table S4** Spearman correlation coefficients of NH_4_NO_3_-extractable PTMs and H^+^ concentration from the revitalization (2^nd^) experiment. Significant correlations are indicated.

|  | Pb | Cd | Zn | H^+^ |
| --- | --- | --- | --- | --- |
| Pb | 1.000 | 0.986* | 0.991* | 0.366 |
| Cd | 0.986* | 1.000 | 0.969* | 0.268 |
| Zn | 0.991* | 0.969* | 1.000 | 0.477 |
| H^+^ | 0.366 | 0.268 | 0.477 | 1.000 |

**Table S5** Correlation of PTM plant concentrations and H^+^ concentration from the revitalization (2^nd^) experiment. Significant correlations are indicated.

|  | Pb | Cd | Zn | H^+^ |
| --- | --- | --- | --- | --- |
| Pb | 1.000 | -0.388 | 0.608* | 0.250 |
| Cd | -0.388 | 1.000 | -0.116 | -0.524* |
| Zn | 0.608* | -0.116 | 1.000 | 0.410 |
| H^+^ | 0.250 | -0.524* | 0.410 | 1.000 |
